# MRI-Based Brain Signatures of Chemotherapy-Induced Peripheral Neuropathy in Cancer Patients: A Systematic Review and Meta-Analysis

**DOI:** 10.3390/diagnostics16111619

**Published:** 2026-05-25

**Authors:** Ioana Creangă-Murariu, Eliza-Maria Armeanu, Vladimir Poroch, Bogdan-Ionel Tamba, Teodora Alexa-Stratulat, Bogdan Gafton, Mihai-Vasile Marinca, Vlad-Adrian Afrasanie, Diana Maria Puscasu, Matei Ioan Rusu, Iulian Prutianu

**Affiliations:** 1 Advanced Research and Development Center for Experimental Medicine “Prof. Ostin C. Mungiu” (CEMEX), Grigore T. Popa University of Medicine and Pharmacy, 700115 Iasi, Romania; ioana.creanga@d.umfiasi.ro (I.C.-M.); bogdan.tamba@umfiasi.ro (B.-I.T.); teodora.alexa-stratulat@umfiasi.ro (T.A.-S.); rusuioanmatei@gmail.com (M.I.R.); 2 Oncology-Radiotherapy Department, Grigore T. Popa University of Medicine and Pharmacy, 700115 Iasi, Romania; bogdan.gafton@umfiasi.ro (B.G.); mihai.marinca@umfiasi.ro (M.-V.M.); adrian.afrasanie@umfiasi.ro (V.-A.A.); tanasamdiana@gmail.com (D.M.P.); 3 Centre for Translational Medicine, Semmelweis University, 1085 Budapest, Hungary; 4 Department of Medical Oncology, Regional Institute of Oncology, 700483 Iasi, Romania; 5 2nd Internal Medicine Department, Grigore T. Popa University of Medicine and Pharmacy, 700115 Iasi, Romania; vladimir.poroch@umfiasi.ro; 6 Department of Palliative Care, Regional Institute of Oncology, 700483 Iasi, Romania; 7 Department of Medical Genetics, Grigore T. Popa University of Medicine and Pharmacy, 700115 Iasi, Romania; 8 Department of Preventive Medicine and Interdisciplinarity–Public Health and Management, Grigore T. Popa University of Medicine and Pharmacy, 700115 Iasi, Romania; iulian-v-prutianu@d.umfiasi.ro; 9 Department of Medical Oncology, Neolife Medical Center, 700503 Iasi, Romania

**Keywords:** chemotherapy-induced peripheral neuropathy, magnetic resonance imaging, functional connectivity, default mode network, brain biomarkers

## Abstract

**Background**: Chemotherapy-induced peripheral neuropathy (CIPN) is a common, disabling toxicity with no validated biomarkers. MRI-based functional neuroimaging could offer insight into central pain processing and may reveal reproducible brain signatures of CIPN. **Methods**: Following PRISMA 2020 (PROSPERO: CRD420251132102), we systematically reviewed whole-brain MRI studies in adult cancer patients with CIPN. Eligible MRI techniques included task-based fMRI, resting-state fMRI, perfusion MRI, and structural MRI. Data were synthesized through voxelwise activation likelihood estimation (ALE), systems-level region-of-interest (ROI) mapping, and proportion meta-analysis of regional involvement. **Results**: Of 2488 screened records, five observational studies were included. The voxelwise ALE analysis did not identify clusters surviving correction, but dispersed foci appeared within the default mode network (DMN), prefrontal executive cortex, and primary sensorimotor regions, suggesting the engagement of these pain-processing networks. ROI synthesis confirmed consistent alterations in the DMN and executive prefrontal and sensorimotor cortices in CIPN patients compared with controls, while the brainstem/periaqueductal gray and cerebellum were rarely implicated. Proportion meta-analysis further quantified these differences: CIPN patients showed altered involvement in 30% (95% CI 0.16–0.48) of contrasts, with the highest frequencies in the DMN (50%), sensorimotor (33%), and executive prefrontal regions (33%). By contrast, control-higher contrasts were less frequent (10%, 95% CI 0.03–0.27), highlighting CIPN-related increases particularly in self-referential and somatosensory networks. **Conclusions**: Across analytic approaches, CIPN is characterized by reproducible alterations in the DMN and executive prefrontal and sensorimotor networks. These central pain signatures represent promising MRI-based biomarkers for identifying and monitoring CIPN in oncology.

## 1. Introduction

Recent advances in magnetic resonance imaging (MRI), computational neuroimaging, and deep learning have substantially transformed the identification of imaging biomarkers across neurological and oncologic diseases. Modern MRI-based approaches increasingly integrate automated segmentation, quantitative tissue characterization, and machine learning-driven analysis pipelines to improve diagnostic precision and biological interpretation [1].

Chemotherapy-induced peripheral neuropathy (CIPN) is a frequent, dose-limiting toxicity of diverse antineoplastic agents, including taxanes, platinum, and vinca alkaloids, that can persist long after treatment completion and substantially erode quality of life and functional independence in cancer survivors [2]. Pooled estimates indicate high incidence and prevalence, with recent data reporting burdens approaching ~30–40% for painful CIPN after platinum and taxane exposure, underscoring its public health impact in the cancer survivorship era [3]. Beyond sensory symptoms, CIPN occurrence is associated with treatment dose reductions or interruptions, amplifying downstream costs and survivorship inequities [4].

Despite being a significant public health issue, CIPN remains a largely subjective diagnosis, highly dependent on patient reporting and physician estimates. Objective biomarkers that reliably identify CIPN, quantify severity, or forecast trajectories remain limited in routine practice. Current guidelines emphasize symptom management and recommend only a narrow set of evidence-based treatments while stating that no pharmacologic strategy is endorsed for prevention, thus highlighting the absence of mechanism-anchored tools [5]. Multiple candidate biomarker domains have been explored (neurophysiology, quantitative sensory testing, blood-based markers such as neurofilament light, and others), yet none has reached broad clinical uptake due to variability in assay standardization, effect sizes, and external validity across regimens and populations [6]. Consequently, personalized medicine for CIPN, risk stratification before therapy, individualized dose adjustments, and mechanism-targeted interventions during survivorship remain aspirational without robust, reproducible markers that map the road from symptoms to pathophysiology [7].

Converging evidence from the broader pain literature suggests that brain network alterations, especially within the default mode network (DMN), executive control, and sensorimotor systems, constitute promising, mechanistically interpretable biomarkers of ongoing pain processing and central sensitization [8,9]. Available reviews and meta-analyses in this research area focus on non-cancer chronic pain and highlight the potential use of functional neuroimaging for diagnosis, stratification, and monitoring, particularly when combined with modern analytic methods [10]. In CIPN specifically, emerging work suggests that central mechanisms (e.g., altered cortical activity, reduced inhibitory tone, neuroinflammation) are most likely involved, thus justifying the systematic evaluation of brain-based markers alongside peripheral measures [11].

In this context, we conducted the first systematic review and meta-analysis of MRI-based neuroimaging studies in adults with CIPN after cancer treatment. Our goal was to assess whether independent investigations converge on common network-level brain alterations that could serve as candidate, objective biomarkers for CIPN, supporting precision phenotyping, risk stratification, and treatment monitoring in oncology practice and trials.

## 2. Materials and Methods

This review was performed with the aim of synthesizing neuroimaging (MRI) findings related to chemotherapy-induced peripheral neuropathy and chronic neuropathic pain in adult cancer populations. The conduct of the review adhered to the PRISMA 2020 reporting standards (Supplementary Table S1) and to the methodological recommendations provided in the Cochrane Handbook for Systematic Reviews of Interventions [12]. A predefined protocol was prospectively registered on PROSPERO (CRD420251132102), ensuring methodological transparency and reproducibility.

### 2.1. Eligibility Criteria

Studies were considered eligible if they included adult cancer patients with a diagnosis of chemotherapy-induced peripheral neuropathy or chronic neuropathic pain following cancer treatment. Neuroimaging had to rely on whole-brain magnetic resonance-based techniques, such as task-related functional MRI, resting-state functional MRI, or perfusion MRI. Control groups (healthy volunteers or other clinically appropriate control groups without CIPN) were required. The primary outcomes of interest were structural or functional brain alterations, reported either as stereotactic peak coordinates or as anatomical labels derived from whole-brain analyses. Studies that did not include cancer patients with chemotherapy-induced peripheral neuropathy or chronic neuropathic pain following cancer treatment were excluded from the review.

Moreover, to be eligible for the meta-analysis, studies were required to (i) perform whole-brain analyses and report peak coordinates in either the Montreal Neurological Institute (MNI) or Talairach space; (ii) clearly define the contrast of interest, either between-group (patients versus controls) or within-patient associations with symptom severity; and (iii) provide group sizes for each arm in between-group analyses or sample size for correlation analyses. Exclusion criteria included reports limited to region-of-interest analyses without a whole-brain search, studies relying solely on small-volume corrections, investigations that did not provide extractable coordinates, and imaging methods outside MRI-based approaches.

We included all original research articles meeting the eligibility criteria, such as interventional and observational trials, as well as conference abstracts, while excluding reviews, case reports, editorials, commentaries, and opinion pieces.

### 2.2. Search Strategy

A comprehensive search of the literature, with no time restrictions, was carried out on 23 August 2025 across three major electronic databases: PubMed, Embase, and the Cochrane Central Register of Controlled Trials (CENTRAL). The following combination of search terms was used: (Neuropathic Pain[Mesh] OR chronic neuropathic pain OR neuropathic pain) AND (Neoplasms[Mesh] OR Carcinoma[Mesh] OR cancer OR carcinoma OR neoplasm* OR tumor* OR tumour*) AND (Neuroimaging[Mesh] OR Magnetic Resonance Imaging[Mesh] OR neuroimaging OR brain imaging OR MRI). This search resulted in a total of 2488 unique records, distributed as follows: 1150 articles from MEDLINE, 1312 from Embase, and 24 from CENTRAL. The reference lists of all included articles were further checked using citationchaser (Version 2.0, Stockholm Environment Institute, Sweden) [13] on 27 August 2025 to identify eligible articles, with no additional articles included.

### 2.3. Study Identification and Selection

All identified references were uploaded into EndNote (Clarivate Analytics) for duplicate removal, leaving 2293 distinct records. Two reviewers (IC-M, E-MA) independently screened studies in two stages. During the first stage, titles and abstracts were examined for alignment with the eligibility criteria, and those considered relevant underwent full-text evaluation. The level of agreement between reviewers was substantial (Cohen’s κ = 0.89). Any disagreements were settled by discussion, and, when a consensus could not be reached, a third reviewer (D-MP) was involved.

In the full-text assessment stage, each study was evaluated against the predefined eligibility criteria established in the registered protocol. Ultimately, 5 studies fulfilled all requirements and were included in the final synthesis (Cohen’s κ = 1.0). The most common reasons for exclusion at this stage were as follows: populations outside the scope (e.g., non-cancer patients or patients without CIPN), other types of neuroimaging techniques besides MRI, a lack of relevant outcomes, or unsuitable comparator groups.

### 2.4. Data Extraction

For each eligible publication, two reviewers (D-MP, E-MA) independently extracted information about study identifiers, participant characteristics, sample sizes, details of the MRI devices, and clinical details of neuropathic pain. Imaging methodologies (e.g., resting-state versus fMRI) were recorded, as were the analytic approaches used (seed-based connectivity, independent component analysis, etc.). For seed-based designs, the seed region itself was documented but not considered a target region in the systems-level synthesis. We also extracted the type and direction of statistical comparisons (patients > controls, patients < controls, or positive/negative associations with symptom severity), all reported peak coordinates with their anatomical labels and reference space, and, where available, peak-level test statistics to facilitate future sensitivity analyses. At this step, discrepancies were resolved by consensus.

### 2.5. Meta-Analysis

Recognizing both the novelty and methodological diversity of the available studies, we prespecified three complementary analytic strategies to maximize interpretability. First, we applied voxelwise coordinate-based meta-analysis using activation likelihood estimation (ALE) to formally test whether independent investigations converged on common neural loci when comparing CIPN patients with control groups. Second, we conducted a systems-level analysis by grouping the reported findings into predefined ROIs and large-scale functional networks, enabling us to identify convergent patterns of anatomical or network involvement even when exact peak coordinates diverged. Third, we performed a proportion meta-analysis of ROI involvement to provide a quantitative estimate of the frequency with which different regions and networks were implicated across studies and contrasts.

#### 2.5.1. Primary Voxelwise Meta-Analysis: Activation Likelihood Estimation (ALE)

Activation likelihood estimation (ALE) was selected as the primary quantitative approach because it formally tests whether reported peaks from independent studies converge at the voxel level beyond chance. The analyses were conducted in GingerALE, incorporating a total of 11 foci derived from 3 independent datasets [14,15,16], representing 45 participants. When reported in Talairach space, coordinates were converted to MNI using the nonlinear transformation [17]. To maintain consistency, contrasts were coded as CIPN patients compared with controls, defined as healthy volunteers, cancer patients without CIPN, or waitlist groups. CIPN-related changes included both instances where patients showed greater activation or connectivity than controls (e.g., CIPN > healthy controls during heat-pain stimulation, CIPN > control in thalamus-seeded connectivity, CIPN > waitlist control in posterior cingulate connectivity) or decreased activation compared to controls.

For the ALE analysis, each study–contrast pair was treated as a distinct “experiment”. All peak coordinates reported for an experiment were entered together with the corresponding patient sample size. Experiments reporting within-patient associations were modeled separately from those comparing CIPN patients with controls. ALE models each reported peak as the center of a three-dimensional probability distribution that accounts for spatial uncertainty for each study, and statistical significance is determined through permutation testing to assess whether clustering across experiments exceeds chance.

All analyses were performed in the MNI152 reference space (91 × 109 × 91 dimensions, 228,483 within-brain voxels, conservative mask). Random-effects modeling applied Turkeltaub’s non-additive correction with a Gaussian kernel full-width at half maximum (FWHM) of 9.24–9.76 mm. Cluster-level inference was applied at *p* < 0.05, with 800 permutations for increases and 400 permutations for decreases, using an uncorrected cluster-forming threshold of *p* < 0.001 and a minimum cluster size of 1632 mm^3^. Coronal slice overlays were generated in MRIcroGL, with parcellated anatomy displayed across Z-coordinates from −102 mm to +88 mm using multi-color segmentation.

#### 2.5.2. Region-of-Interest (ROI) Synthesis

For each eligible study, brain regions associated with CIPN were systematically extracted from the main text, figures, and Supplementary Materials. Both voxelwise and ROI findings were considered. To enable a cross-study comparison, all reported anatomical regions were mapped into five predefined functional categories based on the established neuroimaging literature: brainstem/periaqueductal gray, cerebellum, default mode network (cingulate and posterior midline cortices), lateral/medial prefrontal cortex (executive network), and primary sensorimotor cortex (S1/M1). Given the small number of available studies and the heterogeneity in imaging paradigms, voxelwise convergence alone could underestimate consistent effects. Therefore, a complementary ROI synthesis was performed to identify broader network-level involvement. Two ROI matrices were generated to visualize the distribution of findings. In the study-level matrix, contrasts from the same publication were merged into a single entry, providing a high-level overview of network involvement across studies. In the experiment-level matrix, each reported contrast was treated as a separate entry, thereby preserving the directionality of effects and imaging modality (task fMRI, resting-state fMRI, perfusion MRI). Directionality was coded as either CIPN > control, indicating that patients with CIPN showed greater values than controls (e.g., hyperactivation, hyperconnectivity, or increased perfusion), or CIPN < control, indicating lower values in CIPN relative to controls (e.g., reduced connectivity, lower perfusion, or decreased gray matter density).

#### 2.5.3. Proportion Meta-Analysis of ROI Involvement

To complement the voxelwise and systems-level approaches, we also conducted a proportion meta-analysis of ROI involvement. This method quantified how frequently each predefined region or network was implicated across the literature. Two types of analysis were performed. In the study-level proportion analysis, each publication contributed a single entry, with all reported contrasts collapsed, thereby preventing the overweighting of studies that reported multiple contrasts and providing a conservative measure of reproducibility. In the experiment-level proportion analysis, each study–contrast pair was treated as an independent experiment, preserving directionality and modality while allowing the assessment of whether effects consistently represented CIPN-related increases or decreases. This proportion-based analysis provided a complementary perspective by quantifying the frequency with which different brain networks were implicated across the literature. The study-level analysis emphasized consistency across independent investigations, while the experiment-level analysis retained mechanistic detail by distinguishing between CIPN-related increases and decreases.

### 2.6. Risk of Bias

The risk of bias was assessed independently by two reviewers (IC-M, D-MP) using the Cochrane Risk of Bias tool, ROBINS-I, for observational trials [18]. Any disagreements in judgment were discussed, and unresolved cases were adjudicated by a third reviewer (E-MA).

## 3. Results

**Figure 1:**
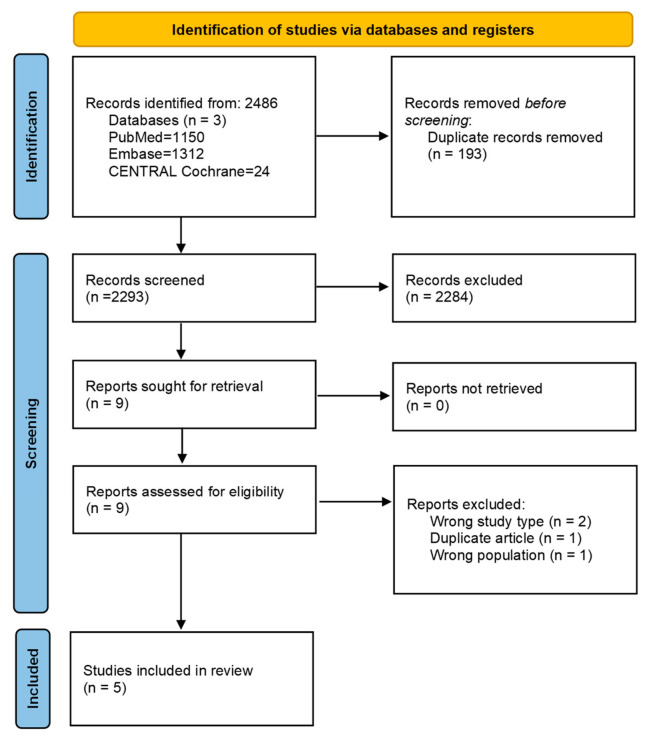
PRISMA flowchart.

The systematic search yielded 2488 records. After screening and eligibility evaluation, five studies enrolling 45 patients fulfilled the inclusion criteria and were incorporated into the final analysis [14,15,16,19,20]. An overview of the study selection process is illustrated in Figure 1.

### 3.1. Characteristics of Included Studies

For the meta-analysis, only four articles were eligible. The studies were conducted in patients with breast cancer, although the composition of the study populations varied; details are presented in Table 1. Boland (2014) and Liu (2022) compared CIPN patients to either healthy volunteers or cancer patients without neuropathy [14,15], Nudelman (2016) longitudinally assessed women with breast cancer who developed CIPN after chemotherapy [19], and Smith (2021) examined breast cancer survivors with CIPN who participated in a behavioral intervention [16]. In terms of methodology, all studies used MRI-based approaches but with different emphases: Boland et al. employed task fMRI during thermal pain stimulation [14], Liu et al. used resting-state fMRI to evaluate thalamus-seeded functional connectivity [15], Smith et al. examined posterior cingulate connectivity with resting-state fMRI [16], and Nudelman et al. combined arterial spin labeling perfusion imaging with voxel-based morphometry to track changes across three timepoints [19]. Details are reported in Table 2. The reported outcomes also differed: Boland et al. focused on altered central pain processing [14], Liu et al. and Smith et al. examined connectivity changes in large-scale networks [15,16], and Nudelman et al. demonstrated associations between CIPN symptoms, altered cerebral perfusion, and gray matter density loss [19]. Boland et al. complemented patient-reported outcomes with neurophysiological testing, whereas the other studies relied primarily on validated self-report measures [14].

### 3.2. Mapping Clusters into Common Functional Networks with ROI

ROI analysis was performed to harmonize heterogeneous neuroimaging findings by mapping reported clusters into common functional networks. This approach enabled us to identify consistent patterns of CIPN-related brain alterations across studies while preserving information about the directionality of effects.

**Figure 2:**
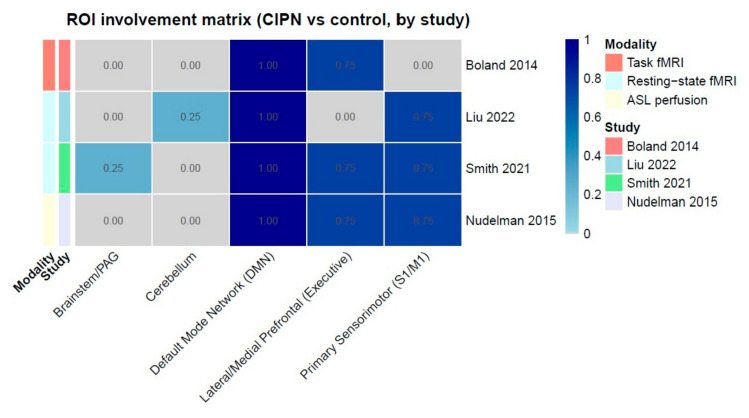
ROI presence matrix across studies of CIPN versus controls. Each row represents one published study, and each column corresponds to a major brain network or region of interest (ROI) [14,15,16,19]: brainstem/periaqueductal gray, cerebellum, default mode network, lateral/medial prefrontal cortex (executive), and primary sensorimotor cortex. Shading intensity (range 0–1) reflects the proportion of ROI involvement reported in that study. This study-level visualization highlights the overall consistency of network-level alterations across the literature, showing the predominant involvement of the prefrontal, cingulate/default mode, and sensorimotor regions in chemotherapy-induced peripheral neuropathy (CIPN).

Across the included neuroimaging studies, we identified the consistent involvement of higher-order cognitive and pain-processing regions. The study-level ROI presence matrix (Figure 2) summarizes these findings by merging contrasts within each study. At this aggregated level, the lateral/medial prefrontal cortex (executive network), the cingulate/default mode network, and the primary sensorimotor cortex emerged as the most consistently involved regions. By contrast, the brainstem/periaqueductal gray and cerebellum were reported less frequently. This pattern suggests that CIPN predominantly engages cortical networks involved in pain modulation, attention, and motor control.

**Figure 3:**
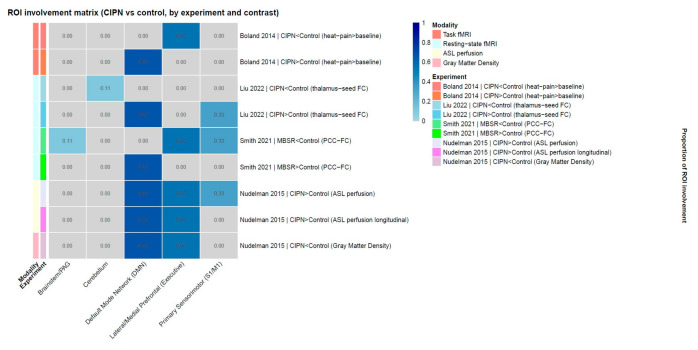
ROI presence matrix across study contrasts of CIPN versus controls. Each row represents an individual experimental contrast (e.g., CIPN > Control or CIPN < Control), and columns represent the same major brain networks/ROIs as in Figure 2 [14,15,16,19]. Shading intensity (range 0–1) indicates the proportion of ROI involvement. Contrast labels specify whether the effect reflected a CIPN-related increase (greater activation, perfusion, or connectivity) or a CIPN-related decrease (lower values relative to controls). This contrast-level visualization complements the study-level matrix by providing mechanistic detail regarding effect directionality and illustrating the heterogeneity between experiments.

To provide further granularity, the experiment-level ROI matrix (Figure 3) displays results for each individual contrast. This approach reveals that, while prefrontal and cingulate involvement was common across studies, the direction of effect differed: some contrasts reported CIPN-related increases (e.g., hyperperfusion or hyperconnectivity), whereas others identified decreases (e.g., reduced connectivity or gray matter density). For example, Boland et al. [14] reported altered central pain processing in CIPN patients using task fMRI, whereas Liu et al. [15] demonstrated both increased and decreased thalamic-seeded resting-state connectivity in different networks. Smith et al. [16] contributed data from a non-CIPN intervention (MBSR), further highlighting variability in comparator conditions.

### 3.3. Activation Likelihood Estimation (ALE) Meta-Analysis

**Figure 4:**
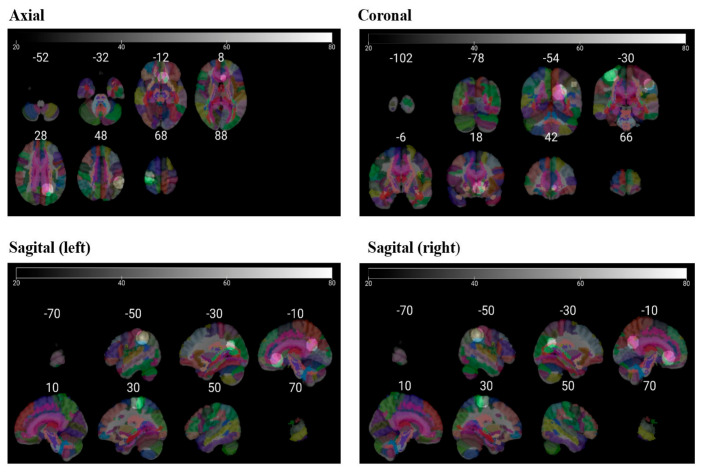
ALE meta-analysis of CIPN versus controls. Axial, coronal, and sagittal views display the results of the activation likelihood estimation (ALE) analysis. No clusters survived cluster-level inference (*p* < 0.05, corrected), with dispersed foci observed across regions of the default mode network (precuneus, inferior parietal lobule, anterior cingulate/medial prefrontal cortex), sensorimotor cortices (precentral and postcentral gyri), executive prefrontal areas (superior frontal gyrus), brainstem (pons), and cerebellum. The color scale reflects ALE values, with warmer colors (yellow–red) indicating higher modeled convergence across studies and cooler colors (blue–green) representing lower values. The light circular outlines mark the individual foci entered into the meta-analysis, corresponding to reported peak coordinates from the included experiments. These results indicate limited voxelwise convergence across experiments, despite the involvement of canonical pain- and motor-related networks in individual studies.

ALE analysis was conducted to formally test whether peak coordinates from independent studies converged on common neural loci beyond chance (Figure 4). This approach provided voxel-level precision in identifying spatially consistent patterns of CIPN-related brain alterations across experiments.

No significant clusters were identified in the ALE analysis (volume > threshold = 0 mm^3^), with ALE scores ranging from 1.4 × 10^−45^ to 0.0084 and minimum *p*-values between 5.09 × 10^−6^ and 1.94 × 10^−5^ but none surviving cluster-level inference. Heterogeneity was negligible (I^2^ = 0.0%, τ = 0, *p* = 0.8646 across models). Visual inspection of the coronal slices showed dispersed foci without robust convergence, with peaks distributed across regions of the default mode network (precuneus, inferior parietal lobule, anterior cingulate/medial prefrontal cortex), sensorimotor cortices (precentral and postcentral gyri), and executive prefrontal areas (superior frontal gyrus), as well as the brainstem (pons) and cerebellum. These findings indicate that, although individual studies implicated several canonical pain- and motor-related regions, the voxelwise analysis did not identify consistent clusters across experiments.

### 3.4. Quantitative Proportion Analysis of Neuroimaging Alterations Across ROI Analysis

**Figure 5:**
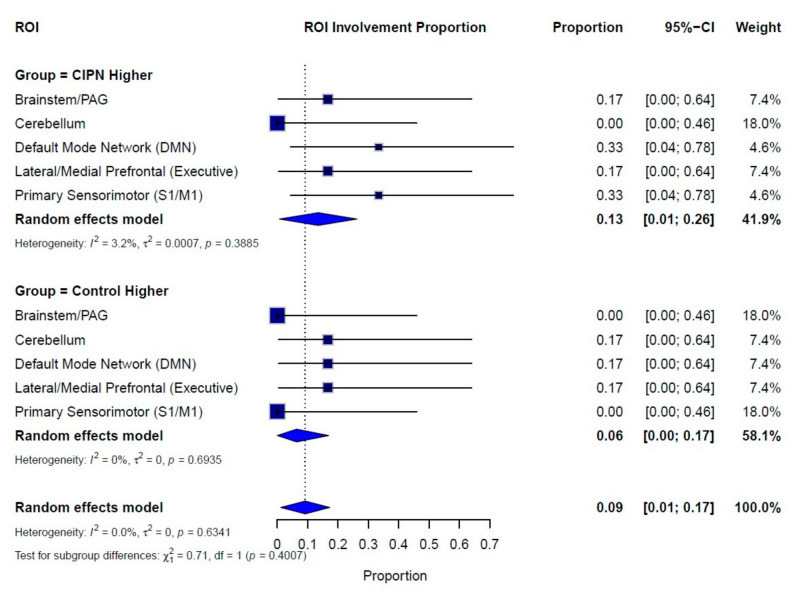
Experiment-level proportion meta-analysis. Forest plot shows the pooled proportion of reported neuroimaging alterations across predefined brain regions of interest. Data are subgrouped into CIPN higher or control higher.

We analyzed proportions at two levels. At the experiment level, every contrast from a study was counted separately, which shows detailed patterns of hyper- and hypoactivation but risks overweighting studies with many contrasts (Figure 5).

**Figure 6:**
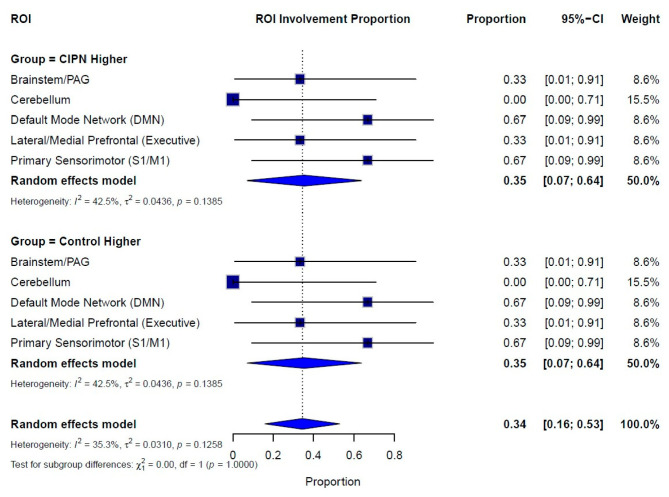
Study-level proportion meta-analysis. Forest plot showing the pooled proportion of reported neuroimaging alterations across predefined brain regions of interest. Data are subgrouped into CIPN higher or control higher.

At the study level, all contrasts were merged into a single entry per study, giving a more conservative picture of consistency across the literature (Figure 6).

When each study–contrast pair was considered independently, the overall pooled proportion of ROI involvement across the five predefined networks was 0.30 (95% CI: 0.16–0.48), indicating that approximately one-third of experimental contrasts identified significant alterations between CIPN patients and controls. Among the individual regions, the default mode network (0.50, 95% CI: 0.12–0.88) exhibited the highest proportion of reported effects, followed by the primary sensorimotor cortex (0.33, 95% CI: 0.04–0.78) and lateral/medial prefrontal regions of the executive network (0.33, 95% CI: 0.04–0.78). In contrast, the brainstem/periaqueductal gray and cerebellum were less frequently implicated (both 0.17, 95% CI: 0.00–0.64).

Stratification by contrast direction further revealed distinct patterns. For CIPN-higher contrasts, the pooled proportion was 0.20 (95% CI: 0.09–0.38), with the default mode and sensorimotor networks emerging as the most consistently represented. Notably, these regions were absent in the control-higher subgroup, suggesting a possible CIPN-specific signature involving self-referential processing and somatosensory integration—processes commonly linked to central sensitization in chronic neuropathic pain. On the other hand, control-higher contrasts yielded a lower pooled proportion of 0.10 (95% CI: 0.03–0.27), characterized by the scattered involvement of the cerebellum, executive prefrontal areas, and portions of the DMN. These findings may indicate relative hypoactivation in CIPN, potentially reflecting chemotherapy-related disruptions of executive and motor circuits.

When contrasts were collapsed within each study, the overall pooled proportion of ROI involvement increased to 0.60 (95% CI: 0.35–0.81), indicating that the majority of independent studies reported at least one significant regional alteration. Across networks, the default mode network (0.67, 95% CI: 0.09–0.99) was the most consistently represented, followed closely by the executive prefrontal cortex (0.67, 95% CI: 0.09–0.99) and the primary sensorimotor cortex (0.67, 95% CI: 0.09–0.99), whereas the brainstem/periaqueductal gray (0.33, 95% CI: 0.01–0.91) and cerebellum (0.33, 95% CI: 0.01–0.91) were implicated less frequently and with greater uncertainty.

Directional stratification yielded balanced pooled proportions for CIPN-higher and control-higher contrasts (0.40 each), suggesting that alterations were observed with comparable frequency in both directions. In the CIPN subgroup, the DMN and sensorimotor regions again emerged as prominent, supporting their roles as candidate CIPN-related networks; however, their recurrence in the control subgroup indicates that these same regions may also undergo relative hypoactivation in some studies. Compared with the experiment-level analysis (overall pooled proportion 0.30, 95% CI: 0.16–0.48), the study-level approach thus revealed more consistent ROI-level involvement across independent datasets. At both levels, the DMN, prefrontal executive regions, and sensorimotor cortex emerged as the most frequently implicated networks, whereas the brainstem/PAG and cerebellum were less reliably observed.

### 3.5. Risk of Bias Assessment

**Figure 7:**
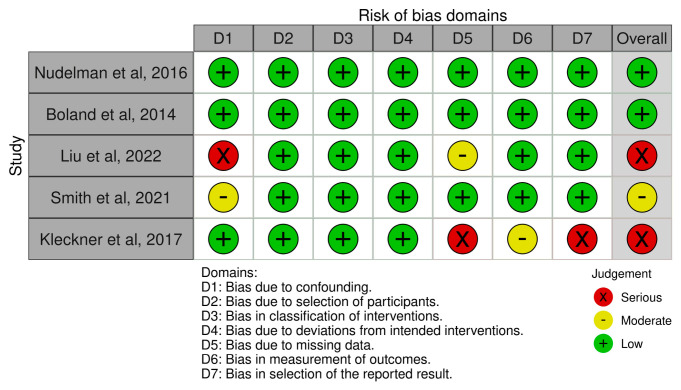
Risk of bias assessment using ROBINS-I across seven domains [14,15,16,19,20]. Green indicates low risk, yellow moderate risk, and red a serious risk of bias.

Overall, the methodological quality of the included studies was mixed (Figure 7). Most domains, such as participant selection, classification of interventions, and deviations from intended interventions, were consistently rated at a low risk of bias. However, concerns were more frequent in specific areas, such as bias due to confounding and missing data. These emerged as recurrent issues, with some studies judged at moderate and others at serious risk. Additional concerns were noted in the measurement of outcomes and the selection of reported results, which contributed to higher overall risk ratings. In contrast, domains such as participant selection and intervention classification were largely well controlled.

## 4. Discussion

This systematic review and meta-analysis synthesized neuroimaging evidence of CIPN in adult cancer patients and identified specific alterations across higher-order cortical networks. Despite the methodological heterogeneity and limited sample sizes, the consistent involvement of the default mode network (DMN), executive prefrontal cortex, and primary sensorimotor cortex emerged, whereas the brainstem/periaqueductal gray and cerebellar regions were less reliably implicated. Although the voxelwise ALE meta-analysis did not reveal significant clusters at a corrected threshold, both the systems-level mapping and proportion analyses highlighted reproducible network-level patterns, suggesting that the absence of voxelwise convergence reflects study diversity rather than a lack of true central effects. Importantly, the absence of significant ALE clusters should not be interpreted as evidence against central nervous system involvement in CIPN. Coordinate-based meta-analytic techniques such as ALE are highly dependent on the number of independent experiments, the sample size, and the spatial consistency of reported peaks. In the present review, only three datasets with 11 total foci were eligible for voxelwise synthesis, substantially limiting the statistical power.

From a clinical perspective, these findings are important because they provide evidence for candidate brain biomarkers of CIPN. The recurrent engagement of the DMN, prefrontal, and sensorimotor systems aligns with established models of chronic neuropathic pain, in which the maladaptive reorganization of pain-processing and motor circuits contributes to persistent symptoms [21]. These network-level abnormalities may serve several roles in oncology care: first, as objective markers that complement patient-reported outcomes and peripheral assessments for the more accurate identification and phenotyping of CIPN; second, as monitoring tools for disease burden and progression, given that perfusion and morphometric studies demonstrate dynamic changes associated with chemotherapy exposure and neuropathy severity [22]; and third, as responsive endpoints in clinical trials, since prior work shows that connectivity and structural alterations in these same networks can normalize following non-pharmacologic interventions such as mindfulness-based stress reduction or exercise [21].

Importantly, the presence of CIPN-related alterations, both in areas where CIPN increases activity and in those with decreased activity, suggests that the disorder is not characterized by a uniform change in brain activity. Rather, the evidence points to heterogeneous patterns of reorganization, with some circuits exhibiting compensatory hyperactivation and others reduced engagement [23]. This bidirectional involvement is consistent with models of central sensitization in neuropathic pain, which posit dysregulated coupling between DMN, executive, and sensorimotor systems as a driver of symptom persistence [24]. The reproducibility of these findings across independent datasets, particularly at the study level, strengthens the confidence that they reflect CIPN-related signatures rather than incidental or modality-specific effects.

Although both CIPN-higher and control-higher contrasts involved overlapping networks, these findings should be interpreted cautiously. Directionality may be influenced by the chemotherapy type, cumulative exposure, symptom duration, pain chronicity, and time since treatment completion [25]. Increased activity may reflect compensatory hyperactivation, whereas decreased activity may indicate impaired network integration [26].

Beyond oncology-specific considerations, an important question is whether the central alterations observed in CIPN are fundamentally distinct from those reported in chronic pain populations without cancer or whether they reflect shared, transdiagnostic mechanisms of persistent neuropathic pain. Neuroimaging studies of chronic neuropathic and non-neuropathic pain consistently demonstrate dysregulation within the default mode network, executive prefrontal regions, and sensorimotor cortices, mirroring the network-level patterns identified in CIPN [27,28]. This overlap suggests that cancer status per se may not uniquely determine the direction or localization of central reorganization; rather, the persistence and neuropathic quality of pain appear to be key drivers of maladaptive brain plasticity. Nevertheless, CIPN arises in the context of systemic chemotherapy, inflammation, and psychological stressors related to cancer diagnosis and treatment, which may modulate the magnitude, temporal evolution, or reversibility of these brain changes [29]. Thus, CIPN may best be conceptualized as a subtype of chronic neuropathic pain that shares core central sensitization mechanisms with other chronic pain conditions, while remaining biologically and clinically distinct due to its oncologic context and treatment-related triggers. Importantly, CIPN remains fundamentally a peripheral neuropathy driven by chemotherapy-related peripheral nerve injury [30]. Therefore, the neuroimaging alterations identified in this review should not be interpreted as primary pathological drivers but rather as downstream or modulatory central nervous system responses associated with chronic pain processing, neuroplasticity, and central sensitization.

An additional challenge relates to the substantial methodological heterogeneity across the included studies. Imaging approaches ranged from task-based fMRI and resting-state connectivity analyses to perfusion MRI and morphometric assessments. Differences in preprocessing pipelines, statistical correction methods, and control populations may also have contributed to the variability in the reported findings.

A key strength of this review lies in its comprehensive and methodologically rigorous approach. We systematically searched multiple databases without time restrictions, applied predefined eligibility criteria, and followed the PRISMA and Cochrane guidelines to maximize transparency and reproducibility. In addition, we employed three complementary analytic strategies—voxelwise ALE, systems-level ROI synthesis, and proportion meta-analysis—which allowed us to capture both fine-grained spatial convergence and broader network-level patterns. This type of design enhances the confidence in the observed findings, since cortical networks such as the DMN, prefrontal, and sensorimotor systems emerged consistently across methods and analytic levels. Another strength is the focus on whole-brain MRI studies only, which minimized reporting bias from region-of-interest approaches and increased the comparability of the results across investigations.

Nonetheless, several limitations must be acknowledged. First, the evidence base remains small, with only a handful of eligible studies and modest sample sizes, limiting the statistical power and contributing to the absence of significant ALE clusters. Second, heterogeneity in study design, imaging paradigms (task-based, resting-state, perfusion, morphometry), and comparator groups (healthy volunteers versus cancer controls) likely reduced voxel-level convergence and complicated the interpretation of directionality. Third, all included studies were observational, with risks of confounding, missing data, and variable outcome measurement, as reflected in the ROBINS-I assessments. Several studies were affected by moderate or serious risks of bias related to confounding, incomplete outcome data, and selective reporting. Factors such as psychological distress, analgesic use, and variability in chemotherapy exposure were not consistently controlled and may have influenced the reported neuroimaging findings. Fourth, the exclusive inclusion of breast cancer populations restricts the generalizability to other malignancies and chemotherapy regimens, while publication bias cannot be excluded given the novelty of the field. The proportion meta-analysis should be interpreted as an exploratory and descriptive analysis rather than definitive evidence of biomarker validity. To reduce the influence of studies reporting multiple contrasts, we performed both experiment-level and study-level analyses, with the study-level analysis collapsing contrasts within each publication. Finally, neuroimaging alone provides an incomplete picture of CIPN pathophysiology; the absence of integrated peripheral measures such as nerve conduction, quantitative sensory testing, or histopathology limits the ability to link central alterations to peripheral nerve injury.

Looking ahead, future research should aim to combine neuroimaging with peripheral assessments, such as quantitative sensory testing, neurophysiological measures, and skin biopsy, to provide a more comprehensive picture of the relationship between peripheral nerve injury and central network reorganization. Moreover, prospective interventional trials are warranted to determine whether baseline alterations or longitudinal changes in DMN, prefrontal, and sensorimotor connectivity can serve as predictors of treatment response or as mechanistic mediators of therapeutic benefit.

## 5. Conclusions

Despite variability in design and methodology, alterations in the DMN and executive prefrontal and sensorimotor networks emerged as promising candidate MRI-based markers of CIPN in cancer patients. These findings support the potential involvement of central mechanisms in chemotherapy-induced neuropathy, while emphasizing that the current evidence remains exploratory due to the small number of available studies and methodological heterogeneity. Future prospective studies using standardized imaging protocols and integrated peripheral assessments are needed to validate these brain-based signatures and clarify their clinical utility for the identification, stratification, and monitoring of CIPN.

## Figures and Tables

**Table 1:** Baseline characteristics table.

Intervention									Control				
Author, Year	Inclusion Criteria	Exclusion Criteria	No. Patients	Age (Mean ± SD)	Range	Duration of Pain	Pain Assessment	Baseline Paine Value (Mean ± SD)	No. Patients	Age (Mean ± SD)	Range	Pain Assessment	Baseline Pain Value (Mean ± SD)
Nudelman et al., 2016 [19]	Breast cancer patients with stage I–III disease, treated with standard-dose, taxane-based chemotherapy regimens	Metastatic disease, prior malignancy, substance abuse, and medical, neurological, or psychiatric conditions that could influence brain structure or function	18	49.4 ± 7.9	NR	13 months	FACT/GOG-Ntx	0.46 ± 0.9;	19	52.0 ± 9.1	NR	FACT/GOG-Ntx	1.09 ± 2.8
Liu et al., 2022 [15]	Breast cancer patients, at least one-year post-therapy, experiencing CIPN for ≥3 months	Cognitive decline, brain metastases, history of psychiatric disorders, ongoing central pain treatment within the past month, severe comorbid illnesses, prior neurological disorders or dementia, inability to complete testing or MRI, and unwillingness to participate	20	52.25 ± 6.55	NR	5.05 ± 0.95 months	VAS	5.65 ±1.66	20	49.95 ± 6.06	NR	VAS	0
Boland et al., 2014 [14]	Myeloma patients previously treated with thalidomide, bortezomib, or vincristine and experiencing neuropathic pain for ≥6 months	Major neurological or psychiatric disorders, MRI contraindications, claustrophobia, left-handedness, and neuropathy from other medical causes	12	63	56–67	2 years	Numeric Rating Scale (NRS), Total Neuropathy Score, reduced version (TNSr), Chronic Pain Acceptance (CPAQ) and Catastrophizing (PCS) questionnaires	Foot: 7.0 [7.0–8.0]; Thigh: 8.0 [7.6–8.0]	12	53	35–58	NRS	Foot: 7.5 [6.6–8.0]; Thigh: 7.7 [5.9–8.0]
Kleckner et al., 2017 [20]	Breast cancer patients with CIPN from chemotherapy with taxane, platinum, and vinca alkaloid	NR	19	50 ± 9 years	NR	NR	CIPN-20-Sensory	>10 on CIPN-20-Sensory	31	50 ± 9 years	NR	CIPN-20-Sensory	0
Smith et al., 2021 [16]	Breast cancer survivors with CIPN	NR	13	52.78 ± 10.77	37–81	NR	7-day Brief Pain Inventory (short form)	>4 (BPI)	10	52.78 ± 10.77	37–81	7-day Brief Pain Inventory (short form)	0

**Table 2:** fMRI acquisition devices and particularities.

Author, Year	Equipment, Model	fMRI Software	fMRI Acquisition Protocol	fMRI Data Processing and Analysis
Nudelman et al., 2016 [19]	Siemens Tim Trio 3T whole-body magnetic	Statistical parametric mapping software SPM8	Q2TIPS pulsed ASL with PICORE labeling; high-resolution T1 MPRAGE and EPI scans for reference/normalization; T2 and FLAIR for pathology screening.	Perfusion-weighted time series generated by control–label subtraction; quantitative perfusion maps created, resampled to 2 mm^3^ voxels, smoothed (6 × 6 × 8 mm). Gray matter density from MPRAGE processed per prior methods; analyses limited to chemotherapy group.
Liu et al., 2022 [15]	Philips Ingenia 3T MR scanner	Data Processing Assistant for rs-fMRI (DPARSF)	Resting-state fMRI; TR = 2000 ms, TE = 30 ms, 36 slices, slice thickness 4 mm, voxel size 2 × 2 × 2 mm^3^.	Preprocessing (motion correction, normalization, smoothing 8 mm FWHM, band-pass filter 0.01–0.08 Hz). Seed-based connectivity using thalamus and PAG. FWE cluster correction.
Boland et al., 2014 [14]	Achieva 3.0T, Philips Healthcare, Best, Holland	Statistical parametric mapping software SPM5	Task fMRI during noxious heat stimulation; TR = 3000 ms, TE = 35 ms, 35 slices, voxel size 1.8 × 1.8 × 4 mm^3^.	GLM with boxcar regressors; within- and between-group contrasts; FWE voxel- and cluster-level correction.
Kleckner et al., 2017 [20]	NR	NR	Resting-state fMRI at 3 timepoints (pre-surgery, 1M post-chemo, 1Y post-chemo).	Seed-based connectivity (posterior insula, anterior cingulate cortex); group comparisons by CIPN-20 sensory scores.
Smith et al., 2021 [16]	3.0-T Siemens TRIO	Data Processing Assistant for rsfMRI (DPARSF) and SPM12 in MATLAB 2017a	Resting-state fMRI; TR = 3000 ms, TE = 34 ms, 38 slices, voxel size 1.6 × 1.6 × 3 mm^3^. Structural T1 MPRAGE also acquired.	Preprocessing (motion correction, normalization, smoothing 4 mm, band-pass 0.01–0.08 Hz, nuisance regression). Seed-to-voxel FC with PCC seed; paired *t*-tests (within group) and two-sample *t*-tests (between groups); cluster-level FWE correction.

## Data Availability

No new data were created or analyzed in this study.

## Supplementary Materials

The following supporting information can be downloaded at https://www.mdpi.com/article/10.3390/diagnostics16111619/s1. Table S1. PRISMA Checklist.

## Abbreviations

The following abbreviations are used in this manuscript:

ALE	Activation Likelihood Estimation
CENTRAL	Cochrane Central Register of Controlled Trials
CI	Confidence Interval
CIPN	Chemotherapy-Induced Peripheral Neuropathy
DMN	Default Mode Network
FWHM	Full Width at Half Maximum
fMRI	Functional Magnetic Resonance Imaging
I^2^	Heterogeneity Index
MBSR	Mindfulness-Based Stress Reduction
MeSH	Medical Subject Headings
MNI	Montreal Neurological Institute (space)
MRI	Magnetic Resonance Imaging
PAG	Periaqueductal Gray
PRISMA	Preferred Reporting Items for Systematic Reviews and Meta-Analyses
PROSPERO	International Prospective Register of Systematic Reviews
ROBINS-I	Risk of Bias in Non-Randomized Studies of Interventions
ROI	Region of Interest
rs-fMRI	Resting-State Functional Magnetic Resonance Imaging
S1/M1	Primary Sensorimotor Cortex (Somatosensory/Motor Cortex)
τ (Tau)	Between-Study Variance
